# The past determines the future: sugar source history and transcriptional memory

**DOI:** 10.1007/s00294-020-01094-8

**Published:** 2020-07-19

**Authors:** Poonam Bheda, Antonis Kirmizis, Robert Schneider

**Affiliations:** 1 Institute of Functional Epigenetics, Helmholtz Zentrum München, Neuherberg, Germany; 2 Epigenetics Lab, Department of Biological Sciences, University of Cyprus, Nicosia, Cyprus; 3 German Center for Diabetes Research (DZD), Neuherberg, Germany; 4 Faculty of Biology, Ludwig-Maximilians Universität München, Munich, Germany

**Keywords:** Transcriptional memory, Single-cell, Inheritance, Chromatin, *GAL*, Microfluidics, History-dependent behavior

## Abstract

Transcriptional reinduction memory is a phenomenon whereby cells “remember” their transcriptional response to a previous stimulus such that subsequent encounters with the same stimulus can result in altered gene expression kinetics. Chromatin structure is thought to play a role in certain transcriptional memory mechanisms, leading to questions as to whether and how memory can be actively maintained and inherited to progeny through cell division. Here we summarize efforts towards dissecting chromatin-based transcriptional memory inheritance of *GAL* genes in *Saccharomyces cerevisiae*. We focus on methods and analyses of *GAL* (as well as *MAL* and *INO*) memory in single cells and discuss the challenges in unraveling the underlying mechanisms in yeast and higher eukaryotes.

## Introduction

In eukaryotes, DNA is packaged into chromatin, and it is the structure of chromatin that is ultimately permissive or restrictive for gene expression. Factors that affect chromatin structure therefore have a profound impact on transcription, and these factors are important to adapt chromatin structure and hence gene induction or repression during changing environmental conditions. Here we will focus on the role of chromatin on heritability of adaptive memory mechanisms in *Saccharomyces cerevisiae* as a response to repeated environmental fluctuations. In this so-called transcriptional reinduction memory, cellular response to an initial stimulus differs from subsequent exposures to the same stimulus, e.g. by affecting the delay or rate of gene induction.

**Fig. 1:**
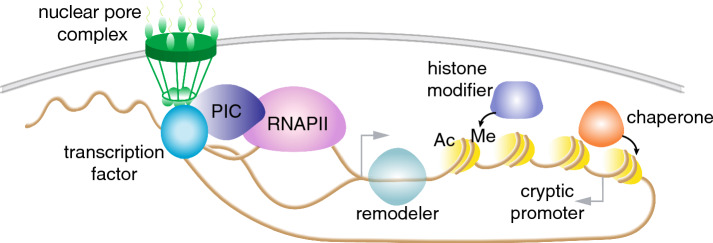
Potential sites for storing chromatin-based memory in *S. cerevisiae*. Components of the transcriptional machinery, such as transcription factors, pre-initiation complex (PIC), and RNA polymerase II (RNAPII), that are recruited during gene induction might remain associated with chromatin even after the stimulus is removed, poising for reinduction via shorter delays. Structural changes in chromatin that occur during induction that can be maintained once the gene is no longer transcriptionally active can also result in transcriptional memory. In *S. cerevisiae,* there are reports of looping of induced genes and translocation to the nuclear periphery that affect reinduction. In addition, effects on nucleosome occupancy perhaps due to remodeler or chaperone activity or covalent histone modifications [e.g. acetylation (Ac) and methylation (Me)] associated with active transcription may also affect reinduction kinetics. *S. cerevisiae* has a compact genome and highly pervasive transcription, where multiple cryptic transcripts are expressed near or in coding regions, including antisense transcripts that can affect the probability of transcribing the coding sequence and could also play a role in transcriptional memory

In Fig. 1, we summarize potential mechanisms for storing chromatin-based memory in *S. cerevisiae*. Although it is easy to conceptualize “where” memory can be stored, how these altered chromatin structures and associations are maintained once the inducing stimulus disappears is difficult to unravel. An even greater challenge to address is whether and how such chromatin alterations can be maintained in mother cells through cell division, in a way that daughter cells can inherit these alterations as well as the associated capacity for transcriptional memory, without prior exposure to the initial stimulus. This requires not only observing single cells as they undergo repeated inductions but also simultaneously tracing cell lineages through divisions. We have recently reported a study using new methods and analyses to tackle the above challenges and decipher the role of chromatin-associated factors in the maintenance and inheritance of galactose-induced transcriptional memory in *S. cerevisiae* (Bheda et al. 2020).

## Sugar source history determines *GAL* transcriptional memory

**Fig. 2:**
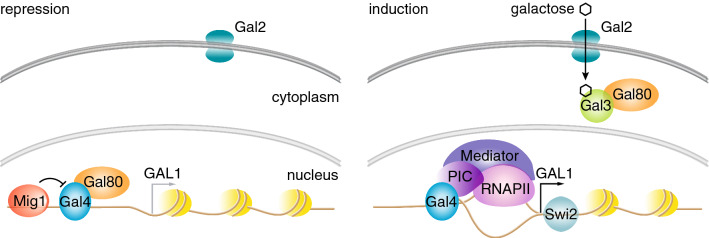
Simplified *GAL* network. (left) In the absence of galactose, Gal80p inhibits Gal4p, the transcription factor for several *GAL* network proteins. Furthermore, the presence of glucose leads to several mechanisms that repress *GAL* genes, including transcriptional repression and active degradation of Gal4p as well as binding of glucose-dependent proteins such as Mig1p to sequences upstream of the *GAL* genes, inhibiting Gal4p binding. (right) In the absence of glucose and when galactose is available, Gal4p levels are increased and Gal3p binds to and sequesters Gal80p away from inhibiting Gal4p. Gal4p binds to the upstream activating sequence (UAS), recruiting the chromatin remodeler Swi2p, which results in the removal of promoter nucleosomes. Consequently, the preinitiation complex (PIC), RNA polymerase II (RNAPII), and Mediator are recruited, leading to transcription of galactose-metabolizing enzymes including Gal1p, Gal10p, and Gal7p, and the transporter Gal2p. The expression of *GAL* genes is tightly controlled by a number of feedback loops, leading up to a 1000-fold increase in mRNA copy numbers under inducing conditions. This process occurs much faster during reinduction, leading to transcriptional memory of *GAL1*, *GAL10*, and *GAL7*

Although *S. cerevisiae* prefers glucose as a carbon source, in the absence of this sugar, it has metabolic networks that allow for the use of alternative energy sources such as galactose. Figure 2 summarizes the central components of the galactose (*GAL*) metabolism network. Once *GAL* genes have been induced with galactose, they retain memory of this induction, and they are primed such that a subsequent exposure to galactose leads to their faster reinduction. *GAL* memory seems to be quite complicated, as both cytoplasmic and nuclear factors contribute to memory, and their relative contributions have been controversial (Kundu and Peterson 2010; Sood et al. 2017; Zacharioudakis et al. 2007). Even mutants that have been described to affect transcriptional memory at the chromatin level under some conditions do not replicate their phenotypes in other conditions. There may be some differences due to observing *GAL1* tagged at the endogenous or an ectopic site or in comparison to a *GAL1* promoter driving fluorescent protein expression, etc.

Validating results and comparing published data is further complicated by the fact that *GAL* memory protocols have varied greatly in the composition of the media (especially the sugar carbon source for repression) as well as the time of exposure to a particular media (i.e. lengths of inductions and repressions), which can significantly affect the role of various factors (Table 1) (Bheda et al. 2020; Brickner et al. 2007; Cerulus et al. 2018; Kundu et al. 2007; Stockwell and Rifkin 2017; Tan-Wong et al. 2009; Zacharioudakis et al. 2007; Zhou and Zhou 2011). Although the effect of some factors appears to be robust between different types of media changes (such as Swi2, Set3, and Cit1) (Bheda et al. 2020; Cerulus et al. 2018; Kim et al. 2012; Kundu et al. 2007), we and others have found that the effect of some factors is less consistent (such as Set1 and Htz1) (Bheda et al. 2020; Brickner et al. 2007; Kundu et al. 2007; Kundu and Peterson 2010; Laine et al. 2009; Zhou and Zhou 2011). This might be due to differences in media composition, media change protocols, or lengths of induction observation, which are not standardized (Table 1). For example, in Zhou et al. the memory experiment began with long-term growth in repressive glucose medium, whereas Kundu et al. initially grew the cells in a neutral raffinose medium, which results in two distinct promoter chromatin conformations (Kundu et al. 2007; Zhou and Zhou 2011). In Bheda et al., we chose to grow cells long-term in raffinose and then repress for the same length of time with glucose prior to each induction in order to keep the initial repression level as similar as possible to the repression between inductions (Bheda et al. 2020). In addition, while some protocols used rich media (YP), we and others have used synthetic media (SC) to minimize background fluorescence. We also used galactose/raffinose mixtures for induction, to avoid memory effects due to fitness instead of *GAL* memory, whereas other protocols used galactose alone for the inductions, or a mixture of galactose and another neutral sugar such as sucrose.

**Table 1:** *GAL* reinduction memory protocols

References	Reporter	Readout	Pre-induction media	Induction media	Intermediate repression media	Reinduction media
Bheda et al. (2020)	Endogenous *GAL1* tagged with GFP	Protein fluorescence by imaging or RNA by RT-qPCR	o/n SC + 2% raffinose, 4 h SC + 2% glucose	1.5–3 h SC + 1.5% galactose/1.5% raffinose	4 h SC + 2% glucose	1.5–3 h SC + 1.5% galactose/1.5% raffinose
Brickner et al. (2007)	Endogenous *GAL1* locus with lacO array or none	Protein fluorescence by staining and imaging GFP-LacI or RT-qPCR		SC + 2% galactose	12 h SC + 2% glucose	SC + 2% galactose
Cerulus et al. (2018)	None or endogenous *GAL1* tagged with yECitrine	Lag time or protein fluorescence by imaging or flow cytometry		2 o/n YP + 5% galactose	2–12 h YP + 5% glucose	YP + 5% galactose
Kundu et al. (2007)	None	RNA by northern blot	o/n YP + 2% raffinose/0.2% sucrose	2 h YP + 2% raffinose/0.2% sucrose	1 h YP + 2% glucose/0.2% sucrose	2 h YP + 2% raffinose/0.2% sucrose
Laine et al. (2009)	*GAL1* promoter driving *SEN1*	RNA by RT-PCR	2% glucose to mid-log phase	2.5 h 2% galactose	0.5 h 2% glucose	2% galactose
Stockwell et al. (2017)	Endogenous *GAL1* tagged with yECerulean	Protein fluorescence by imaging		24 h SC + 2% galactose	12 h SC + 2% glucose	24 h SC + 2% galactose
Sood et al. (2017)	Endogenous *GAL1* locus with lacO array or mCherry	Protein fluorescence by staining or live-cell imaging of GFP-LacI or flow cytometry		o/n SC + galactose	12 h SC + glucose	0.5–10 h SC + galactose
Tan-Wong et al. (2009)	*GAL1* promoter driving *FMP27*	RNA by RT-qPCR	o/n YP + 2% glucose	1 h YP + 2% galactose	1 or 4.5 h YP + 2% glucose	2 h YP + 2% galactose
Zacharioudakis et al. (2007)	Endogenous *GAL1* tagged with GFP	Protein fluorescence by flow cytometry or imaging		24 h YP + 2% galactose	12 h YP + 2% glucose	YP + 2% galactose
Zhou et al. (2011)	None	RNA by RT-qPCR	glucose	2.5 h galactose	1 h glucose	galactose

It is perhaps not surprising that media differences lead to such variability when considering that the chromatin state between inductions, during which the gene retains memory (i.e. memory window), might be greatly affected by the level of the initial induction. Moreover, the initial induction itself is closely dependent on the initial state of repression. For instance, long-term growth in glucose results in a much more repressed state than growth in the less repressive raffinose, and thus growth in the former results in a slower first induction. Such differences in induction levels could potentially obscure the effects exhibited by some mutants. In addition, the duration of induction is also an important consideration, where at early timepoints perhaps differences in induction levels are not so apparent. Given the differences in media change protocols and especially the extent of repression by glucose in various studies, it has been difficult to fully reconcile the importance of previously identified regulators. This truly points to the fact that future gene expression is highly dependent on history.

In addition to *GAL*, transcriptional memory has been described for the *MAL* genes as well as *INO1* in *S. cerevisiae*. The *MAL* genes are responsible for respiratory metabolism of another alternative carbon source, maltose, and memory of a previous induction appears to work similarly mechanistically to *GAL* memory (Cerulus et al. 2018). *INO1* memory is rather different to *GAL* and *MAL* memory. Ino1p, an enzyme involved in inositol biosynthesis, is induced upon inositol starvation. Unlike *GAL* and *MAL* genes, however, expression of *INO1* is lower in subsequent inductions than in an initial induction (Brickner et al. 2007). As more studies are performed using these systems it will be interesting to determine whether variations on media protocols will also have different effects such as in the case of *GAL*.

## Using single-cell assays and live-cell observations with lineage information to dissect maintenance and inheritance of memory

**Fig. 3:**
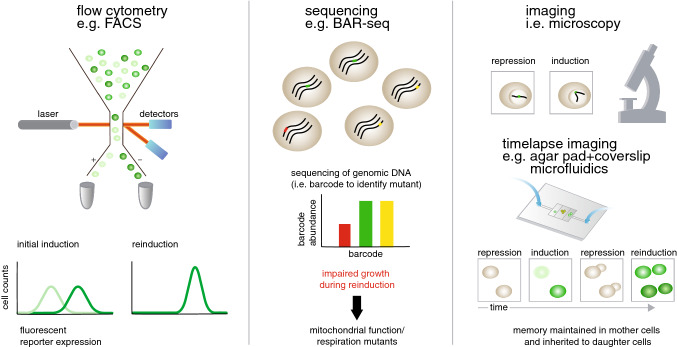
Three methods to analyze single-cell transcriptional memory in yeast: flow cytometry, sequencing, and imaging. (left) Flow cytometry is used to analyze single cells by granularity, size, or fluorescence. Galactose-naïve cells display bimodal Gal1p expression at either intermediate galactose concentrations or intermediate timepoints during induction (transient bimodality), whereas reinduced cells with transcriptional memory have a unimodal distribution of Gal1p expression. (middle) BAR-seq can identify the number of mutant cells at a given timepoint by reads of their genomic DNA barcodes. Mitochondrial function and respiratory mutants have slow lag times until escaping growth arrest during *MAL* and *GAL* memory. (right) Imaging by microscopy is used for both fixed and live single cells. *GAL1* remains localized to the nuclear periphery after induction, but unlike *INO1* this localization does not contribute to memory. To facilitate timelapse imaging, cells are captured in chambers made by agar pads and coverslips or more recently in microfluidic chambers. *GAL1* memory is maintained in single mother cells and inherited to daughter cells. For more details, see main text

Despite the caveats with confounding effects and differences between protocols, there seems to be some consensus in the field that at least under certain conditions, chromatin plays a role in transcriptional memory mechanisms. Immediately this raises the questions as to how long these altered chromatin structures survive beyond an initial induction, and whether they are actively maintained (replicated) through cell division. Although we can acquire an initial understanding of these processes using population-based measurements, to truly dissect these mechanisms it is imperative to observe single cells exposed to repeated stimuli as they proceed through cell division. However, the majority of studies on transcriptional memory in yeast have so far been limited to bulk measurements of transcripts historically by northern blotting or more recently by RT-qPCR. The first step towards understanding inheritance of memory has been to observe this phenomenon in single cells. For this, three main techniques have been used so far: flow cytometry, sequencing, and imaging (Fig. 3).

Acar et al. and Biggar et al. used flow cytometry to observe how expression of either a *GAL1* promoter-driven fluorescent protein or Gal1p fused to a fluorescent protein, respectively, is distributed based on the history of previous inductions/conditions (Acar et al. 2005; Biggar and Crabtree 2001). These studies revealed that there can be significant variability in an initial induction leading to bimodal expression depending on the galactose concentration due to feedback loops. Zacharioudakis et al. and Sood et al. showed that transcriptional memory reduces this variability, allowing the population to have a uniform response during reinduction (Sood and Brickner 2017; Zacharioudakis et al. 2007). Sood et al. further used fluorescence-activated cell sorting (FACS) to identify a Gal1p mutant that specifically affects memory by disrupting its interaction with Gal80p.

Cerulus et al. carried out a screen to identify *MAL* memory mutants by using barcoding in combination with sequencing to allow recovery of single-cell information (BAR-seq) to calculate individual mutant lag times for growth (i.e. time until cells escape growth arrest due to changing carbon source) by genomic DNA reads (Cerulus et al. 2018). Interestingly, they identified mutants of mitochondrial function and respiration with impaired *MAL* memory, and these mutants were also found to have impaired *GAL* memory. Previously, single-cell RNA-sequencing has had limited use in yeast due to difficulty with cell wall degradation and the low number of transcripts per cell; however the adaptation of droplet-based single-cell RNA sequencing by in-droplet spheroplasting increased RNA capture efficiency, and therefore this method may also be used for future single-cell transcriptional memory studies (Jariani et al. 2020).

Imaging has been extensively employed for single-cell analyses of *GAL* transcriptional memory. Sood et al. and Brickner et al. used imaging by integrating reporters at the *GAL1* and *INO1* loci and following their nuclear localization by staining or live-cell imaging (Brickner et al. 2007; Sood et al. 2017). This revealed that both *GAL1* and *INO1* translocate to the nuclear periphery upon induction, and that these genes remain associated with the nuclear envelope even after the inducing stimulus is removed. The association with the nuclear envelope in the case of *INO1* promotes reinduction expression, whereas this localization does not seem to contribute to *GAL1* memory (Light et al. 2013; Sood et al. 2017). Additionally, Zacharioudakis et al. and Cerulus et al. used imaging of heterokarya (cells that have fused their cytoplasms but not their nuclei) to explore the necessity of cytoplasmic components on *GAL* and *MAL* transcriptional memory, respectively (Cerulus et al. 2018; Zacharioudakis et al. 2007).

Even though the above studies have been valuable in understanding transcriptional memory in yeast, they lacked time-resolved analyses of the same cells over time and rather relied on analyzing different cells taken at different timepoints from a single culture that is undergoing the memory protocol. The reason for this is that these techniques rely on fixation/lysis of cells or simply lack of feasibility. To address memory maintenance and inheritance, it is necessary to utilize live-cell methods that allow observation of this process over time. Multiple groups used time-lapse microscopy to monitor transcriptional memory in single live cells. Recently, Cerulus et al. captured cells between an agar pad and a coverslip to observe that cells arrest growth when shifted from glucose to galactose, and that the lag time until cells resume growth during reinduction is dependent on the length of glucose-mediated repression, which interestingly follows the expression of proteins involved in respiration (Cerulus et al. 2018).

Although time-lapse microscopy with captured cells was a step forward for following memory inheritance in single mother-daughter pairs, the above setup does not allow cells to be followed through media changes. Thus, Stockwell et al. used a microfluidics device for automated media changes and observed transient bimodality and unimodality in levels of Gal1p from populations of single cells during galactose induction and reinduction, respectively (Stockwell and Rifkin 2017). We recently implemented a high-throughput microfluidics setup in combination with a time-lapse imaging-based screening for analyzing hundreds of mutants on a single-cell level by observing fluorescence from tagged Gal1p as the strains were subjected to repeated inductions (Bheda et al. 2020). With microfluidics it is possible to follow single cells over time, registering gene expression, cell division and cell growth, among other phenotypes. However, with the limited time resolution in Stockwell et al. and during our screen, it was not possible to capture cell divisions, and therefore lineage information was lost.

Since our aim was to establish the inheritance and potential epigenetic nature of memory, it was necessary to observe the same cells over time as they undergo cell divisions. To accomplish this, we additionally integrated a microfluidics setup that allowed us to observe the same cells throughout the entire memory experiment. We tracked individual cells and their progeny by collecting microscopy images with sufficient time resolution such that a custom-made segmentation and tracking software could identify the same cells across multiple images. With the aid of a nuclear reporter, we could also define the lineages of the cells. Our data finally allowed us to address the question of inheritance of transcriptional memory in *S. cerevisiae* in single cells (Fig. 3) (Bheda et al. 2020).

To date, the only other experiment that we are aware of that broaches the question of transcriptional memory inheritance used elutriation as a technique to split the cell population into mother and daughter cells (Kundu et al. 2007). This technique relies on centrifugation while simultaneously flowing liquid or gas in the opposite direction to separate cells based on size, shape, and density. Mother cells that were previously induced were allowed to undergo one division, then mother and daughter cells were separated by elutriation, and *GAL1* reinduction kinetics were analyzed in the mother and daughter subpopulations by northern blotting to show that daughter cells did, on average, inherit *GAL* memory. However, using this technique, Kundu et al. were not able to analyze individual cells and consequently their lineages (Kundu et al. 2007).

We used our cell-tracking microfluidics to analyze memory maintenance and inheritance in single mother-daughter pairs to capture the dynamics and variations in transcriptional response that are lost with previously established approaches. Our setup allowed analysis of transcriptional memory inheritance from a single cell to its progeny, with the ability to follow multiple generations separately. This required the development of novel analyses using partial correlations, relative difference, and Bayesian statistics to quantitatively assess memory maintenance in mothers and inheritance in mother-daughter pairs. Importantly, using this analysis we discovered that mother cells establish memory during an initial induction, permitting higher gene expression in a subsequent induction due to a shorter delay in transcriptional activation. We also observed that this memory is inherited in single-mother daughter pairs, whereas specific mutants can disrupt memory transmittance to result in asymmetric memory inheritance (Bheda et al. 2020). We anticipate that these analyses will be applied to future studies of transcriptional memory in yeast.

## Anticipated transcriptional memory mechanisms in other yeasts and higher eukaryotes

To create a positive memory system at the chromatin level in *S. cerevisiae* where a gene is expressed faster or earlier in a subsequent induction requires either the continued binding of a chromatin regulator to the memory locus, or a mechanism to overcome the off/repressive state (Fig. 1). Intriguingly, the more recently diverged *Saccharomyces uvarum* species does not display *GAL* transcriptional memory, as it already expresses a higher basal level of Gal1p in glucose conditions, leading to rapid *GAL* expression already during an initial induction as a result of positive feedback (Sood and Brickner 2017). Although this is beneficial for this specific organism in adapting to changes from glucose to galactose, it comes at a fitness trade-off for the species.

In *S. cerevisiae* the most repressive chromatin conformation is achieved by full nucleosome occupancy and unmodified lysines maintained by the sirtuin family of histone deacetylases. Even linker histone Hho1 (H1 homolog) does not appear to play a major role in genome organization and chromatin structure (Panday and Grove 2017). Therefore, *S. cerevisiae* is limited to acquiring transcriptional memory by events that occur during transcriptional activation—i.e. nucleosome loss, active histone marks, and maintenance of chromatin regulators in cis. Notably, *S. cerevisiae* lacks specific methyltransferases that incorporate “repressive” histone modifications such as H3K9me and H3K27me. The repertoire for potential chromatin-based transcriptional memory mechanisms (especially repressive ones) is significantly increased already in the yeast *Schizosaccharomyces pombe*, which has additional pathways for affecting chromatin structure by some repressive histone modifications and RNA interference, where overcoming these barriers to transcription in a first induction and not reestablishing them during repression could also lead to memory during reinduction. Further layers of chromatin regulation are found in metazoans, such as DNA modifications, enhancers, and other gene regulatory elements, all associated with specific histone modifications not present in *S. cerevisiae*. We anticipate that extrapolating our memory inheritance analyses to yet undiscovered transcriptional memory systems and in higher eukaryotes will lead to exciting avenues of research.
